# Multidisciplinary management of a fused maxillary incisor: Case report with 5‐year follow‐up

**DOI:** 10.1002/ccr3.3629

**Published:** 2020-12-10

**Authors:** Rania M. El Backly, Gehan Sherif Kotry, Hassan Moussa

**Affiliations:** ^1^ Endodontics Conservative Dentistry Department Faculty of Dentistry Alexandria University Alexandria Egypt; ^2^ Periodontology Department of Oral Medicine and Periodontology Faculty of Dentistry Alexandria University Alexandria Egypt; ^3^ Orthodontics Department of Orthodontics Faculty of Dentistry Alexandria University Alexandria Egypt

**Keywords:** dental fusion, endodontics, interdisciplinary studies, orthodontics, periodontics

## Abstract

Detailed treatment planning and execution are crucial if regenerative approaches are to be attempted to retain fused permanent teeth. Long‐term follow‐up is necessary to monitor the stability of the final outcome, both esthetically and functionally.

## Graphical abstract

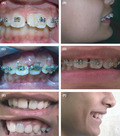

## 1 INTRODUCTION

A 12‐year‐old male presented to the dental clinic with a supernumerary tooth fused to a maxillary central incisor. Treatment included an interdisciplinary regenerative approach with 5‐year follow‐up. Fusion cases require meticulous diagnosis and treatment planning with the main objective of retaining the tooth and obtaining a satisfactory outcome.

“Twinning anomalies” are abnormalities of tooth shape. They include gemination, concrescence, megadontia, macrodontia, and fusion. Gemination occurs when two teeth develop from one tooth germ, resulting in a large tooth, and the number of teeth is normal. Usually, the division is incomplete and the tooth presents a single root and canal.^1^ On the other hand, fusion arises by the union of two normally separated tooth germs that can be either complete or incomplete. In the case of union of two permanent teeth, the patient presents with one large tooth and an incomplete dentition. When fusion is between tooth germs of a normal tooth and a supernumerary tooth, the number of teeth is complete and it becomes more challenging to differentiate fusion from gemination. As the term “double‐tooth” was introduced,^1^ it presented various management challenges for the clinician particularly if they include anterior teeth. The most common problem is esthetics, not only because of their abnormal shape and size, but also because of subsequent orthodontic problems especially crowding. Caries and periodontal complications may also be at play when optimal plaque control is hindered by a subgingival fissure or union lines.^2^, ^3^

Different approaches have been proposed to deal with cases of “double teeth” ranging from surgical separation followed by esthetic recontouring only,^4^ or accompanied with endodontic treatment followed by orthodontic treatment as well.^3^, ^5^ A recent systematic review of 72 cases ^6^ showed that most of these cases required interdisciplinary management. Half of the cases were subjected to restorative treatment following surgical hemisectioning that did not always include removal of the sectioned fragment. Most of these cases required orthodontic management to achieve a satisfactory esthetic outcome. Several cases were subjected to extraction followed by prosthetic options, while some cases were left untreated.

Interestingly, even with multidisciplinary management, the approach in each discipline may vary tremendously from cases treated conservatively using vital pulp therapy procedures to cases requiring complete pulpectomies and root canal treatment.^5^, ^7^, ^8^ Regarding the surgical approach following hemisectioning, the option to use grafting materials and membranes has not always been pursued although several reports have recommended their use to minimize bone loss, hence better periodontal outcome.^2^, ^9^

The current case report of a young male documents an unconventional multidisciplinary approach to the management of a double maxillary anterior fusion requiring orthodontic treatment. The CARE guidelines were implemented in reporting of this case (www.care‐statement.org).^10^

## 2 CASE PRESENTATION

### 2.1 Patient Information

A twelve‐year‐old male patient presented to the Conservative Dentistry Department outpatient clinic, Faculty of Dentistry, Alexandria University in October 2013, complaining of poor esthetics. The patient was systemically healthy. He had malocclusion that was classified as Class II division 1 and requiring orthodontic treatment. Macrodontia of the maxillary central incisors was evident. Previous dental history included extractions of primary teeth and failed orthodontic treatment.

### 2.2 Clinical findings

**Figure 1:**
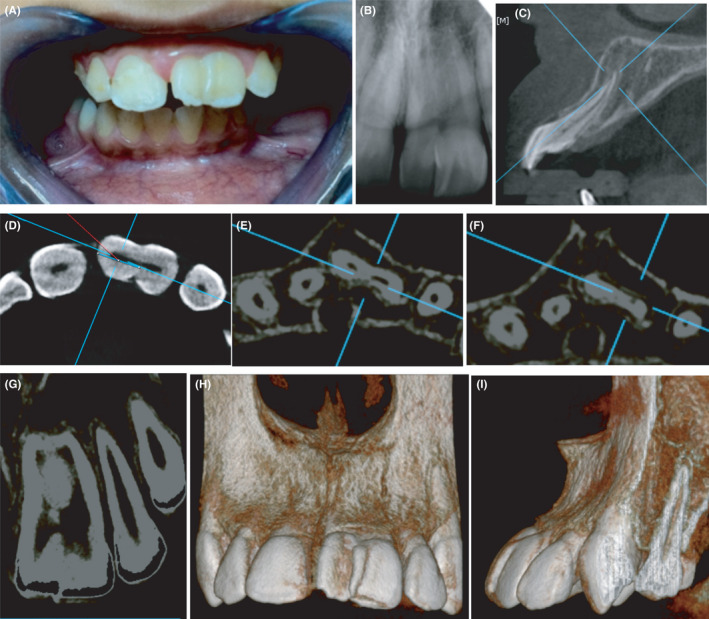
Pre‐operative assessment of the case showing a 12‐yr‐old male patient presenting with malocclusion classified as class II division I and requiring orthodontic treatment. Macrodontia of the left maxillary central incisor is evident. Patient was systemically healthy and his chief complaint was poor esthetics. (A) Pre‐operative frontal view showing a maxillary left “double‐tooth” where the crown of the maxillary left central incisor is fused with a supernumerary tooth; (B) Digital periapical radiograph of the tooth; (C) CBCT sagittal slice showing severe labial proclination of the fused tooth; (D‐F) Axial CBCT slices in the fused tooth at the cervical, middle, and apical thirds of the tooth, respectively. Note the labial opening of the apical foramen; (G) CBCT coronal slice showing the fused tooth with a common root canal system in the coronal up to the middle third followed by separation and rejoining just before the apex; (H) 3D CBCT rendering labial view; (I) 3D CBCT rendering proximal view. To estimate the bone graft amount: the volume of an anticipated cylinder was calculated using the formula : V = hπr^2^ H = 22.83 mm. , R = 3.15 mm. ,V = 22.83 x 3.15x 3.15 x 3.14 = 711. 30 mm^3^ = 0.71130 cubic cm^3^ = 0.7 grams; therefore, approximately 1 gm of graft was calculated to fill the socket

Upon oral examination, it appeared that the crown of the left maxillary central incisor was fused to a mal‐aligned supernumerary tooth (mesiodens) which was situated between the two central incisors (Figure 1A). The teeth were endodontically and periodontally healthy and the patient had fair oral hygiene.

### 2.3 Diagnostic assessment

Digital periapical radiographs revealed that the two teeth were fused from the crown to the end of the root (Figure 1B). Cone beam computed tomography scans (CBCT) evaluation revealed the two teeth were completely fused with a common root canal system in the coronal to mid‐third then followed by two separate canals. At one level palatally, the teeth appeared to have a common root canal system in the apical 12 mm (Figure 1C‐G). In order to pursue orthodontic treatment of the case, a decision was made to retain the maxillary left central incisor following hemisectioning of the fused tooth along with bone graft placement in the resultant socket. The CBCT was valuable in evaluating the clinical condition as well as estimating the amount of required bone substitute (Figure 1C‐I). The treatment plan was discussed with the patient's guardian and as such provided informed written consent according to the requirements of the intuitional review board of the Faculty of Dentistry, Alexandria University (IRB NO: 00 010 556 – IORG: 0 008 839) (https://ohrp.cit.nih.gov/search/search.aspx).

### 2.4 Therapeutic interventions

#### 2.4.1 Endodontic therapy

**Figure 2:**
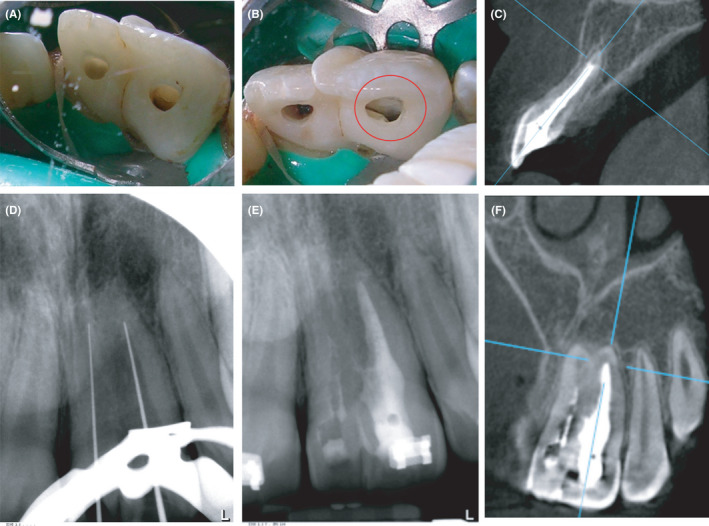
Root canal treatment of double‐tooth prior to surgical sectioning procedures. (A) access cavity preparations in the maxillary left central incisor and fused supernumerary tooth; (B) image showing communicating isthmus between maxillary left central incisor and conjoined supernumerary tooth; (C) showing length of mineral trioxide aggregate (MTA) monoblock filling; (D) working length measurement for the fused tooth; (E) immediate post‐filling periapical radiograph showing MTA monoblock filling. Note the presence of communicating fins between the two teeth; (F) Coronal CBCT slice showing the MTA monoblock filling

Under local anesthesia (2% Mepivacaine, 1:20 000 epinephrine), access cavities were prepared for the fused teeth showing pulpal communication between the teeth (Figure 2A‐B). Working lengths were estimated and the canals were instrumented using the step‐back technique till size 60 hand files along with copious irrigation with 2.5% Sodium Hypochlorite (NaOCl) (Figure 2C‐D). Finally, Ethylenediaminetetraacetic acid (EDTA) (Glyde, Dentsply) was placed for 1 minute to remove the smear layer followed by irrigation with saline. The canal was completely dried using sterile paper points. A thick slurry mix of Pro‐root white Mineral Trioxide Aggregate (MTA) (Dentsply Sirona) was packed using an amalgam carrier and Schilder pluggers. The supernumerary (mesiodens) was irrigated, dried, and filled with Calcium Hydroxide Ultracal XS (Ultradent). The access cavity for the central incisor was then restored with composite (Ivoclar, Vivadent) (Figure 2E‐F).

### 2.5 Surgical procedure

**Figure 3:**
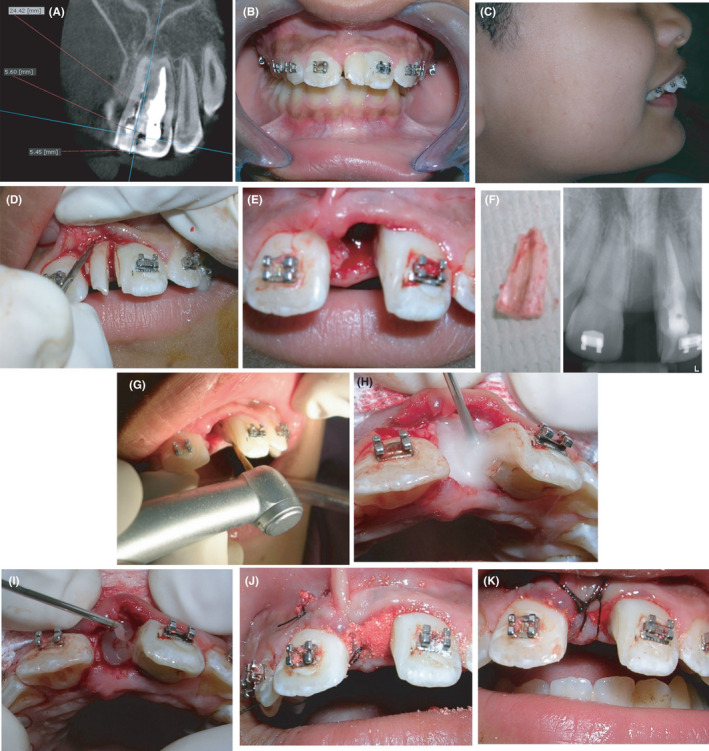
Surgical procedures for sectioning of the fused tooth. (A) Coronal CBCT slice with length measurements to identify surgical plane of sectioning; (B, C) Immediate pre‐operative clinical photos; (D) Use of periotomes for gentle luxation of sectioned supernumerary tooth; (E) socket following removal of supernumerary tooth; (F) extracted sectioned tooth and immediate post‐sectioning periapical radiograph; (G) smoothening of the resected surface; (H) socket treatment with Straumann PREF gel prior to Emdogain^®^ placement; (I) injection of Emdogain® along resected surface and in socket; (J) bioactive glass packed in the socket; (K) socket after grafting and suturing procedures

**Figure 4:**
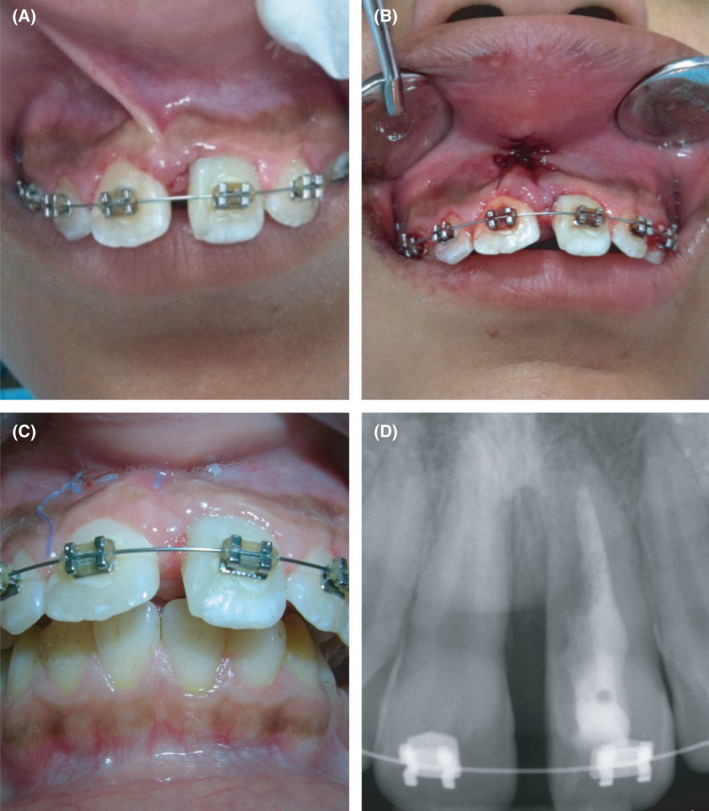
Frenectomy procedures for the patient performed 4 months after the initial surgical intervention. (A) shows high frenal attachment; (B) immediate post‐operative photo after frenectomy procedure with sutures in place; (C) Healing 2 months after frenectomy; (D) Periapical radiograph 2 months following frenectomy procedure

Two months later, the patient was recalled for surgery. The day prior to surgery the patient had orthodontic brackets and bands cemented (Figure 3A‐C). At time of surgery, the patient's face was disinfected , the mouth was rinsed with chlorhexidine HCL (Hexitol, Arab Drug Company ), and the teeth were polished under local and regional anesthesia (infiltration + incisive and infraorbital nerve blocks with Mepicaine‐L (Mepivacaine HCL 2% + levonordefrin 1:20 000, Alex Co. For Pharmaceuticals), sulcular incisions were made mesial, labial, and palatal using micro‐surgical blades (Braun Melsungen AG, Aesculap division, Tuttlingen). A mucoperiosteal flap was reflected just to expose the crestal bone. Using a round end tapered coarse diamond stone (Jota AG, Ruthi, Switzerland) on a high‐speed handpiece with copious cooling, an initial guiding groove was created through the distal 1/3 of the mesiodens to avoid injury to the permanent incisor. Then, the crown was sectioned along that groove till the cervical line (Figure 3D). Periotomes (Kohdent Roland Kohler Medizintechnik GmbH & Co. KG) were then used to dissect the periodontal ligament attachment. A peri‐operative digital periapical radiograph was taken to confirm proper orientation of the cuts before proceeding. Sectioning took place using a cross‐cut tapered carbide extra‐long surgical fissure bur on a bur extender (Jota, AG) mounted on a low speed handpiece at 30 000 RPM and 1:1 torque (Nouvag AG, Goldach) with copious saline irrigation. After gentle luxation, the sectioned part was grasped and removed using artery forceps. The sectioned surface was smoothened and lightly planed with curettes (Figure 3E‐G). The socket was rinsed with sterile saline, and hemostasis was accomplished using sterile gauze in preparation for placement of Emdogain^®^ (Institute Straumann AG Postfach, Basel Switzerland). Pref Gel ™ ( Institute Straumann AG Postfach, Basel Switzerland ) was then injected according to the manufacturer's instructions onto the sectioned and planed surface after which it was rinsed with sterile saline. Emdogain ^®^ was also injected in the socket itself and mixed with the synthetic bone graft (70S/30C Bioglass,^11^ particle size 150‐300 µm) (Figure 3H‐I). The graft was packed into the socket until it was slightly overfilled (Figure 3 J). The flap was then repositioned without tension and sutured over the socket using 5/0 silk sutures (Ethicon, Johnson & Johnson Bridgewater, New Jersey & Cincinnati) (Figure 3K). The patient was given post‐operative instructions. Analgesics and NSAIDs were prescribed, Cataflam 50 mg (Novartis Pharmaceuticals Corporation), Alphintern (Amoun Pharmaceutical Company) as well as antibiotics, Dalacin C 300 mg (Pfizer, Canada) twice daily for five days. At the 4‐month follow‐up, the examination following healing and tissue contraction revealed a high frenal attachment in relation to both central incisors and a decision to perform a frenectomy was taken (Figure 4).

### 2.6 Orthodontic intervention

The patient was referred for orthodontic evaluation after surgical‐endodontic interventions. The decision to follow‐up for one year prior to orthodontic treatment to correct maxillary prognathism was taken to allow for the evaluation of the success of surgical intervention. One year later and for a period of 24 months, the following orthodontic protocol was adapted. Bilateral upper first premolars were extracted followed by full upper and lower arches bonded edge‐wise appliances with pre‐elasticated 0.022x0.028‐inch brackets (Ormco, California, USA). Mini implant between first molars and second premolars in the maxilla was placed for retraction of the upper anterior segment, then leveling and alignment took place with 0.014 and 0.016‐inch NiTi wires (Ormco, California, USA) followed up by 0.016 × 0.022‐inch NiTi wires in the lower arch and 0.016 x 0.022‐inch stainless steel wires in the upper arch. Crimpable hooks (Ormco, California, USA) were placed between the upper laterals and canines and en‐masse retraction was done using very light force. The use of sliding mechanics was chosen to suit the patient's capabilities to follow‐up treatment in our clinic as well as using very gentle forces not exceeding 125 gm in retraction. After full retraction and consolidation of spaces, finishing was done using 0.018 × 0.025‐inch stainless steel wires in the upper arch and 0.019 × 0.028‐inch stainless steel wires in the lower arch. A removable Hawley retainer was used for one year following de‐banding.

#### 2.6.1 Follow‐up until four years

**Figure 5:**
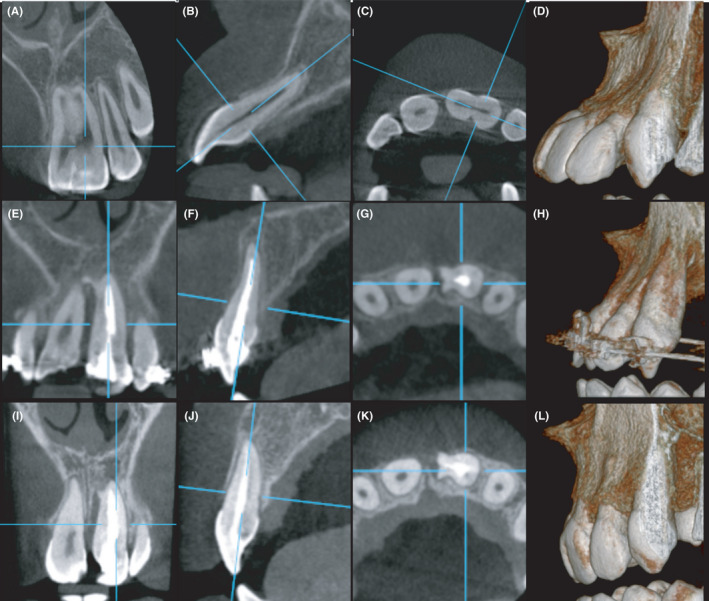
CBCT scans of the patient pre‐operatively in (A‐D), 1 year post‐operative (E‐H) and 4 years post‐operative in (I‐L), where (A, E, I) represent coronal slices; (B,F,J) represent sagittal slices; (C,G,K) represent axial slices; and (D,H,L) represent 3D volume renderings from the profile view

**Figure 6:**
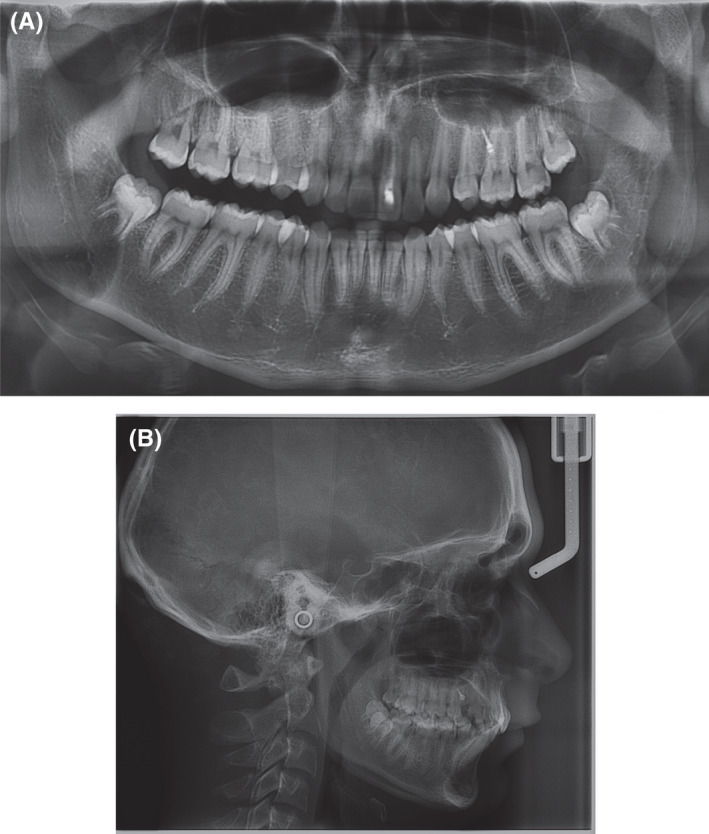
Panoramic and lateral cephalometric radiographs of the patient 4 years post‐operatively in (A) and (B), respectively showing good alignment of the teeth with restoration of normal occlusion

The patient was regularly recalled up to 4 years post‐surgical intervention to ensure the absence of complications and general satisfaction with the ongoing treatment. Surgical re‐intervention was planned in the event of development of any complications. Following endodontic treatment, the patient reported mild sensitivity to cold which quickly resolved within two weeks post‐surgically and the tooth remained asymptomatic. Following surgical intervention, healing was complication‐free. CBCT at one‐year follow‐up revealed the apex of the supernumerary tooth had been retained (Figure 5). Clinical examination revealed presence of a sinus tract draining pus which was traced to the central incisor and was communicating with a deep pocket on the mesial surface of the central incisor. A decision was made to perform conservative treatment via periodontal scaling and curettage to avoid disturbing the tooth movement during active orthodontic treatment. The patient was recalled for bimonthly periodontal examination (Table 1) and remained asymptomatic and stable up to 4 years in spite of persistence of a deep periodontal pocket (Figure 5). The tooth showed no mobility yet some discoloration was the only complaint by the patient. After 4 years, CBCT and periapical radiographs showed the presence of a thin labial plate of bone although a periapical lesion had developed surrounding the left maxillary central incisor and adjoined apex of supernumerary (Figure 6).

**Table 1:** Periodontal measurements throughout the treatment period

Maxillary right central incisor	Probing depth (mm)						Clinical attachment loss (mm)					
	DP	DL	MidP	MidL	MP	ML	DP	DL	MidP	MidL	MP	ML
3 mo	1	2	1	1	2	3	0	0	0	0	0	0
18 mo	2	3	2	3	2	5	0	0	0	1	0	3
48 mo	1	1	1	1	1	5	0	0	0	0	0	3
60 mo	2	2	1	1	1	3	1	1	0	0	0	2

Maxillary left central incisor	Probing depth (mm)						Clinical attachment loss (mm)					
	DP	DL	MidP	MidL	MP	ML	DP	DL	MidP	MidL	MP	ML
3 mo	2	2	2	2	4	5	0	0	0	0	2	3
18 mo	2	3	2	3	5	5	0	2	0	2	3	3
48 mo	2	2	2	2	5	9	1	1	1	1	3	7
60 mo	2	2	2	3	4	5	1	1	1	2	3	4

Abbreviations: DL, distolabial; DP, distopalatal; midL, mid labial; Midp, mid palatal; ML, mesiolabial; MP, mesiopalatal.

#### 2.6.2 Re‐intervention surgery

**Figure 7:**
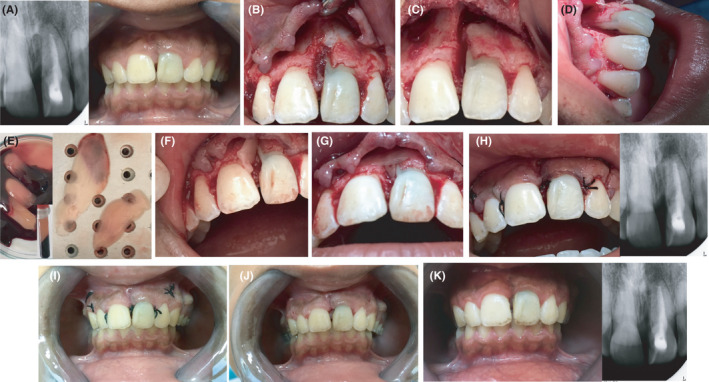
Re‐intervention surgery after 4 years to remove retained apex of the supernumerary tooth and re‐plane the resected mesial surface. (A) Immediate pre‐operative periapical radiograph showing a periapical radiolucent lesion and retained apex of supernumerary tooth (left) and clinical photograph (right); (B) Following flap reflection revealing long vertical defect on mesial surface of maxillary left central incisor; (C, D) Creation of a vertical groove reaching the retained apical segment of the supernumerary tooth; (E) Preparation of Platelet‐Rich Fibrin (PRF) membranes; (F) Planing of root surface and placement of PRF membrane in the vertical groove; (G) Placement of second PRF membrane underneath the flap prior to repositioning; (H) Final clinical image after suturing (left) and post‐operative periapical radiograph (right) showing resection of root end of maxillary left central incisor and shaving off of retained apex; (I) One week after the re‐intervention surgery; clinical image after removal of sutures; (J) clinical image 2.5 months after re‐intervention surgery; (K) Clinical image and periapical radiograph one year after re‐intervention surgery showing periapical healing and deposition of new bone

A decision to re‐intervene surgically was made and initiated following termination of the orthodontic retention stage (Figure 7). This was done by reflecting a full thickness flap which exposed a long vertical narrow osseous defect on the mesial surface of the maxillary left central incisor . Periodontal debridement of the granulation tissue and root planing was done. The retained apex was removed and root end resection of the maxillary left central incisor was also done. Platelet‐rich fibrin (PRF) membranes were prepared from 20 cc of the patient's venous blood without anticoagulant. The blood was centrifuged at 3000 rpm for 10 minutes to produce 3 PRF clots which were then squeezed to obtain compact membranes. One PRF membrane was then adapted along the planed root surface and another membrane was placed underneath the flap immediately prior to its repositioning. The patient was followed up for another year and revealed markedly reduced periodontal probing and attachment loss readings (Table 1) with very good gingival contour. Periapical radiographs revealed periapical bone healing; however, mesial crestal bone height did not improve at the time of last follow‐up.

**Figure 8:**
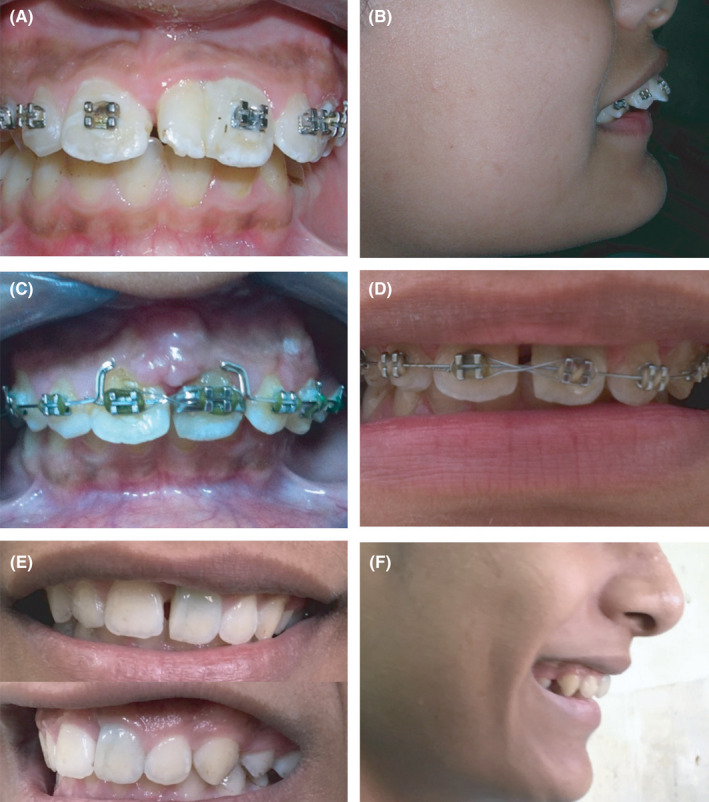
Clinical photographs of the patient at different follow‐up periods. (A, B) Pre‐operative photographs; (C,D) 1 year follow‐up photographs; (E,F) 5 years follow‐up photographs (1 year after the re‐intervention surgery)

Regarding the orthodontic treatment, skeletal changes can be summarized as follows. In the antero‐posterior plane, the discrepancy between maxilla and mandible was reduced due to retruding of the maxilla and protruding of the mandible (as evidenced by: SNA‐ SNB ‐ ANB ‐ Wits appraisal) (Table 2). In the vertical plane, increasing of the facial height occurred (as evidenced by: Fr/MP ‐ SN/MP ‐ Pal/MP). Regarding the dental changes, reduction of overjet occurred mainly by retrusion of the maxillary incisors (as evidenced by: 1 Fr ‐ 1 Md) (Figure 8). After one year of de‐bonding and retention using the Hawley appliance, the patient showed stable occlusion and no morbid changes in the surgery area. At 4‐year CBDCT follow‐up, no signs of root resorption or blunting could be detected. However, 1 year of follow‐up after the second surgery revealed some relapse as evidenced by increased spacing between the maxillary central incisors. Clinical documentation of case progression is shown in Figure 8.

**Table 2:** Skeletal and dental measurements (cephalometric) before and after orthodontic treatment

	Pre‐tr	24 months	36 months	Comments
SNA	82	80	81	Reduction of maxillary prognathism
SNB	75	76	77	Mandibular forward growth
ANB	7	4	4	Normalizing maxillary‐ mandibular relation
SN/MP	28	29	29	Hypodivergent
Fr/MP	14	16	17	Hypodivergent
Pal/M	13	15	15	
Gonial angle	114	116	117	Mandibular growth forward
Facial plane	87	89	90	Mandibular growth forward
1 Frankfurt	117	112	110	Dental of upper incisor
1 NA (degrees)	22	10	12	Dental correction of upper incisor
1 NA (mm)	4	2	2	Reduction of overjet
1 Mand	112	107	105	Up righting of lower incisors
1 NB (degrees)	35	30	28	Dental correction
1 NB (mm)	7	5	4	Reduction in axial inclination
FMIA	54	57	58	Reduction in axial inclination
Wits	7 mm	5mm	4 mm	Correction of basal arch relation

Abbreviations: 1 Fr, upper incisor axial inclination to frank. Plane; 1 Mand, lower incisor axial inclination to mandibular plane; 1 NA, upper incisor axial inclination to line between point A and N; 1 NB, lower incisor axial inclination to line between point N and B; ANB, A point‐nasion‐B point angle (ANB : relation between maxilla and mandible, S : sella tursica, N : nasion); FMIA, Frankfurt mandibular incisal angle; Fr/MP, Frankfurt/mandibular plane; Gonial angle, angle between lower border of the mandible and tangent to the mandibular body; Pal/MP, Palatal/mandibular plane; SN, anterior cranial base, A: represents maxilla; SN/MP, sella‐nasion line/mandibular plane; SNA, sella‐nasion‐A point angle; SNB, sella‐nasion‐B point angle (B : represents mandible); Wits, Witwatersrand analysis.

## 3 DISCUSSION AND CONCLUSIONS

The current case presented with a complete dentition and an abnormally large central incisor. This may be due to the fusion between a permanent central incisor and a supernumerary mesiodens. The term “double‐tooth” therefore seems more applicable in such a case. The main problems encountered in the management of double teeth are the lack of sufficient information reporting the best line of treatment and the relative absence of long‐term follow‐up of these interventions.^3^ Consensus indicates that a multidisciplinary approach is the best choice for dealing with these cases. Therefore, a combined endodontic, periodontal, and orthodontic intervention was applied. The management strategy determined was dependent on clinical and radiographic findings and anticipated topography following hemisectioning of the twinned tooth. The current case reports a five‐year follow‐up period. The main observations were that this protocol resulted in improved function, maintenance of alveolar bone height as well as patient and parent satisfaction with the treatment. Orthodontic results were stable and resulted in a normal occlusion and a drastic improvement in esthetics. There was no root resorption and barely any mobility after the end of therapy. Endodontic therapy using a mineral trioxide aggregate (MTA) monoblock was selected since MTA represents a highly biocompatible material that has excellent sealing properties, in addition to exceptional antibacterial and bioinductive abilities.^12^ MTA monoblock filling has been suggested for filling of immature necrotic teeth with open apices and has been shown not only to strengthen the roots but that upon its removal for post space preparation, its sealing properties appear not to be affected.^12^, ^13^, ^14^ MTA is also most commonly used as a retrograde filling material to seal apical ramifications during endodontic root resection due to its previously mentioned properties.^12^ In the current case report, since endodontic therapy was commenced prior to surgical intervention, it was crucial to select a filling material that would not only maintain long‐term sealing but would also have bioactive properties once it was exposed to the periodontal and bone tissues upon hemisectioning of the fused tooth.^12^ This in addition to the presence of the bone graft material and enamel matrix derivative which would—together with the MTA—potentially provide an excellent substrate for new hard tissue formation and periodontal re‐attachment. The periodontal strategy applied was to try and obliterate the resultant socket from extraction of the mesiodens in order to promote tissue regeneration and preserve bone around the remaining central incisor. Splinting was also applied to prevent tooth movement and stabilize the ensuing clot. The lag period between surgery and commencement of active orthodontic therapy in the current work was to allow these tissues to regenerate uninterrupted. The use of enamel matrix derivative (EMD) in conjunction with the bone alloplast plays a crucial role in orchestrating tissue healing and bone formation. Studies indicate that EMD significantly decreases interleukin‐1 beta (IL‐1β) and RANKL expression, thereby promoting bone remodeling. EMD increases bacterial and tissue debris clearance, as well as fibroplasia and angiogenesis by inducing endothelial cell proliferation, migration, and capillary‐like sprout formation.^15^ Another important aspect of EMD is its reported biomimetic effect and its capacity to play a role in dentin, acellular cementum, and alveolar bone formation during embryonic tooth development.^16^ Therefore, the use of EMD was an attempt to prompt the regeneration of not only new bone, but also of a more favorable periodontal attachment to the hemisectioned tooth. With the same methodology, a reported study showed a higher incidence of healed periodontal ligament tissues (PDL) around re‐implanted teeth in beagle dogs.^17^ Other histologic studies demonstrated that EMD results in limited epithelial down growth. Moreover, human biopsies have reported the possibility of complete periodontal regeneration or new connective tissue attachment after EMD application to roots in intra bony periodontal defects.^18^ Unlike regular periodontal surgery, periodontal measurements at the mesiolabial aspect of tooth #21 at the site of the extracted mesiodens were constantly changing. Three months after surgery, there was a 3 mm CAL which increased to 7 mm at one year. After starting the orthodontic movement, further CAL reached 9mm then stabilized at 7 mm after 4 years. An explanation is the possibility that moving the tooth led to bone and tissue remodeling and hence the increased measurements during active orthodontic movement. Moreover, clinical and radiographic examination revealed the presence of a deep groove running from the cervical aspect of the tooth including a great aspect of the root, probably due to cutting through the root of the mesiodens at a slightly more distal plane. This groove might have predisposed to plaque retention and some attachment loss. Although the deep periodontal pocket that developed may be considered a clinical shortcoming of this case, a decision to delay the second intervention was made to avoid disturbing bone remodeling during the phase of active orthodontic intervention. Additionally, a mid‐line diastema remained again possibly due to the retained root apex which may have prevented closure of this space during orthodontic activation. These sub‐optimal outcomes lead to the decision to perform a second surgery to improve the final outcome which may include orthodontic management again in the future. Other cases studies such as that by Kim et al^19^ while show a better final outcome did not represent the same challenges as found in the current case including the presence of pulpal communication which entailed root canal therapy of the tooth prior to resection and profound treatment planning using multiple CBCT scans. While this case is similar to ours, it appears very difficult to have ideal results due to the many variables involved. A compromise is usually reached, and satisfactory, stable clinical, and esthetic results are considered sufficient by the clinician particularly if the patient is satisfied with the final result. Indeed, the remaining sites at both central incisors were normal and in line with a clinically healthy periodontal attachment. Continuous follow‐up and periodontal maintenance were carried out during the follow‐up period and a second surgical intervention was performed using PRF. The choice to use PRF was done to avoid excessive costs by the re‐use of EMD for the second surgery in addition to the ability of PRF to serve as a membrane to augment the mucoperisoteal flap thereby possibly contributing as well to the overall enhanced results. This is in virtue of its well‐documented benefits in regenerative dentistry applications and oral surgery indications.^20^ Indeed, this lead to further probing depth and attachment loss reduction. Regarding the orthodontic results, they showed improvement and normalization of facial and dental arch relationships due to the use of retraction mechanics in the upper arch with gentle force control, which was chosen to suite the surgical‐endodontic treatment that was performed in the area of the upper central incisors, as well as facilitating proper follow‐up of changes in the periodontally and endodontically treated tooth. During the initial evaluation of the patient, the decision to extract the fused central and supernumerary tooth could have resulted in correction of the overjet and have stable occlusion results, yet it would have jeopardized the chance to have a normally shaped dentition and smile similar to that in the presence of the natural centrals, laterals, and canines. The risk of undergoing retraction of the surgically managed segment was finally evaluated after one‐year follow‐up, since the patient and his family did not accept removal of the fused teeth and could sustain such a prolonged procedure with necessary follow‐up. In conclusion, the collaboration between the different specialties facilitated treatment and tailored the needed protocol for the correction of such a clinically challenging situation.

## 4 PATIENT PERSPECTIVE

After five years, both the patient and his guardian reported satisfaction with the final treatment outcomes, particularly the improvement in both front and profile views. The only mild concern was the discoloration of the tooth and different treatment options were discussed with the patient for the near future.

## CONFLICT OF INTEREST

None declared.

## AUTHOR CONTRIBUTIONS

RE: performed the endodontic treatment and contributed to all surgical interventions and follow‐up, diagnosis and treatment planning and drafted the manuscript; GK: performed the periodontal interventions and all surgical procedures, planning of all interventions and follow‐up, and was a major contributor to writing the manuscript; HM: performed all orthodontic interventions. All authors read and approved the final manuscript.

## ETHICS APPROVAL AND CONSENT TO PARTICIPATE

The patient’s guardian provided informed written consent prior to all interventions according to the requirements of the intuitional review board of the Faculty of Dentistry, Alexandria University (IRB NO: 00010556 – IORG: 0008839) (https://ohrp.cit.nih.gov/search/search.aspx).

## CONSENT FOR PUBLICATION

Informed written consent for publication was obtained from the patient’s guardian.

## Data Availability

Data sharing is not applicable to this article as no datasets were generated or analyzed during the current study.
